# Validation of the SF12 mental and physical health measure for the population from a low-income country in sub-Saharan Africa

**DOI:** 10.1186/s12955-020-01323-1

**Published:** 2020-03-18

**Authors:** Julius Ohrnberger, Laura Anselmi, Eleonora Fichera, Matt Sutton

**Affiliations:** 1 School of Public Health, MRC Centre for Global Infectious Disease Analysis, Imperial College London, London, England; 2 Division of Population Health, Health Services Research & Primary Care, University of Manchester, Manchester, England; 3 Department of Economics, University of Bath, Manchester, England

**Keywords:** SF12 scale validation, LMICs, Mental health, Physical health, Malawi, Health policy

## Abstract

**Introduction:**

The Short Form Survey 12-item (SF12) mental and physical health version has been applied in several studies on populations from Sub-Saharan Africa. However, the SF12 has not been computed and validated for these populations. We address in this paper these gaps in the literature and use a health intervention example in Malawi to show the importance of our analysis for health policy.

**Methods:**

We firstly compute the weights of the SF12 physical and mental health measure for the Malawian population using principal component analysis on a sample of 2838 adults from wave four (2006) of Malawian Longitudinal Study of Aging (MLSFH). We secondly test the construct validity of our computed and the US-population weighted SF12 measures using regression analysis and Fixed Effect estimation on waves four, seven (2012) and eight (2013) of the MLSFH. Finally, we use a Malawian cash transfer programme to exemplify the implications of using US- and Malawi-weighted SF12 mental health measures in policy evaluation.

**Results:**

We find that the Malawian SF12 health measure weighted by our computed Malawian population weights is strongly associated with other mental health measures (*Depression:-0.501, p = < 0.001; Anxiety:-1.755; p = < 0.001*) and shows better construct validity in comparison to the US-weighted SF12 mental health component (*rs = 0.675* versus *rs = 0.495*). None of the SF12 measures shows strong associations with other measures of physical health. The estimated average effect of the cash transfer is significant when using the Malawi-weighted SF12 mental health measure (*treatment effect: 1.124; p = < 0.1*), but not when using the US-weighted counterpart (*treatment effect: 1.129; p > 0.1*). The weightings affect the size of the impacts across mental health quantiles suggesting that the weighting scheme matters for empirical health policy analysis.

**Conclusion:**

Mental health shows more pronounced associations with the physical health dimension in a Low-Income Country like Malawi compared to the US. This is important for the construct validity of the SF12 health measures and has strong implications in health policy analysis. Further analysis is required for the physical health dimension of the SF12.

## Introduction

Improving physical and mental health in populations living in poverty are both important global development goals [5, 17, 24]. The Short Form 12-item Survey (SF12) is a common patient reported instrument to measure physical and mental health related quality of life and widely applied in research on populations from Sub-Saharan Africa [1, 6, 11, 13, 15, 19]. The SF12 health dimensions are computed with factor weights based on a US-population study [9]. Despite its application in several studies, the SF12 has neither been validated for Sub-Saharan populations nor have SF12 dimension weights been computed and tested for these populations [22].

Using a validated health outcome measure is important in all empirical analyses. Using a non-validated SF12 with incorrect population weights (e.g. the commonly applied US-weights) can for example mislead analytical findings and policy implications, as we demonstrate in this study.

Previous research on the validity and population-weights of the SF12 scale for mental and physical health has focused on populations from High-Income Countries. One study by Gandek et al., (1998) for instance finds that for nine European countries there is little difference in using the US-derived weights or the country-specific item-weights. Existing studies that test the validity of the SF12 for Low- and Middle-Income Countries either do not compute country-specific weights or do not compare the country-specific SF12 version to the widely used US- population weighted version [18, 20, 30]. Further are these studies building their analysis on small non-representative population samples limiting the scale of research findings.

Another limitation of the literature is the use of cross-sectional data. It is important to use longitudinal design to make assessment on the temporal stability of the SF12 measures for individuals and to address unobserved heterogeneity in mental and physical health [10].

We contribute to the literature in several ways. Firstly, we compute and validate the SF12 for the Malawian population, a Low-Income Country from Sub-Saharan Africa and produce population norms from the derived measures for the Malawian population. We use the Malawian population because its population characteristics are comparable to populations from other Sub-Saharan African Low-Income Countries [3]. In Malawi, about every second person lives in poverty which is comparable to the average poverty rate of 41% in Sub-Saharan African countries [27, 28]. Individuals are exposed to a high health risk environment with life expectancy at birth just 59 years of age (58 years in Sub-Saharan Africa) and HIV being the main contributor to mortality [29]. Prevalence of mental health problems is high, with about 30% of primary care patients affected by mental health disorders and 19% suffering of depression. These numbers reflect the typical high prevalence of mental health in Sub-Saharan Low-Income Countries which further motivates the choice of the Malawian population for the analysis [12, 23].

Secondly, we use a large population representative sample to compute Malawian population weights and compare the SF12 measures obtained applying the Malawi or the US-population weights. Thirdly, we test the construct validity of these measures and the temporal stability of the SF12 measures. And finally, we show if and how differences between the US- and Malawi-weighted mental health SF12 measure may matter for policy evaluation using the empirical example of a cash transfer programme.

## Material and methods

### Malawian longitudinal study of family and health (MLSFH)

We use the fourth wave (2006) of the Malawian Longitudinal Study of Family and Health (MLSFH), a representative study on the rural Malawian population of Age 15 and older [14], to compute the Malawi-weights for the SF12 health scales. Participants were visited at home and interviewed by a trained interviewer in their local language (Chi Chewa, Chi Yao or Chi Tumbuka). Participants had to consent their involvement in the study at the onset of the interview. The MLSFH population is representative of the rural population in Malawi which was established elsewhere [14]. The survey sample was designed using cluster-randomisation. Seven waves of data exist. The first wave of data was collected in 1998 and the latest in 2012. The sample was updated to represent the initial size and migration follow-up studies were conducted. Previous cohort-analyses showed that attrition does not bias the analytical findings from the MLSFH [14].

The fourth survey round of the MLSFH includes information on important determinants of both physical and mental health such as alcohol consumption, smoking or social activities and environmental risk factors. In order to validate the SF12 Malawi-weighted health scales, we use in addition to the fourth wave, waves seven (2012) and eight (2013) of the MLSFH which collected clinically validated measures of depression (PHQ9) and anxiety (GAD7) [16, 21]. The 2012 and 2013 rounds also included new objective measures of physical health (BMI and average grip strength), useful for testing the construct validity of the physical and mental health domain measures. We use the 2012 and 2013 samples only to test for construct validity as these are constructed as sub-samples of the 2006 sample, including only adults of age 45 + .

We also use information on the Malawi Incentive Programme (MIP) to test if different population weights of SF12 measures have implications for the analysis. The MIP was a one-year (2007–2008) randomised-controlled cash transfer trial in northern, central and southern Malawi. Cash transfers were conditional on keeping the HIV status over the intervention period. Individuals from the fourth wave of the Malawian Longitudinal Study of Family and Health (MLSFH) in 2006 were randomised into the MIP. Wave five (2008) of the MLSFH is included in the analysis of the MIP. Kohler and Thornton [13] provide a detailed discussion of the MIP.

### SF12 measure of physical and mental health

The Short Form 12-item Survey is a general measure for both physical and mental health related quality of life and is computed following the scoring algorithm developed by Ware et al. [25]. The instrument consists of 12 questions with binary and Likert-scale answer options. Of these, six are related to physical health and five are related to mental health. A final question combines both physical and mental health dimensions.

Answers from the 12 questions are then grouped into the following eight functional health subdomains, all standardized to a range 0 to 100: Physical Functioning, Role Physical, Bodily Pain, General Health, Vitality, Social Functioning, Role Emotional and Mental Health.

To compute the respective physical and mental health dimension of the SF12, weights or factor loadings are derived from principal component analysis with a standard two-vector solution of which one factor corresponds to mental health and the other to physical health. The eight standardized health subdomains are multiplied by the factor loadings of the respective health dimensions to compute a factor score. The factor scores are then summed up and set to the mean 50 with standard deviation of ten. The SF12 has a maximum value of 100 indicating best possible mental health and a minimum value of zero.

### Explanatory variables mental health domain

We use the clinically validated General Anxiety Disorder Assessment (GAD7) instrument [21]. The GAD7 consist of seven questions asking the individuals how about the frequency of underlying symptoms of anxiety. The GAD7 ranges from value 0 indicating lowest to 21 indicating the highest possible traces of anxiety.

We also use the clinically validated Personal Health Questionnaire 9-item version (PhQ9) [16]. The PhQ9 detects traces of depressions and consists of nine questions asking the individuals how often he/she was bothered in the last 2 weeks by underlying depressive symptoms. The PhQ9 is computed by summing over the numerically coded responses leading to a scale with minimum value 0 indicating lowest possible traces of depression and a maximum value of 29 indicating the highest possible traces of depression.

A third measure is self-reported subjective wellbeing, ranging from 0 very unsatisfied to 4 very satisfied. The measure is reported in all three waves, and GAD7 and PhQ9 are reported in wave seven and eight of the MLSFH.

### Explanatory variables physical health domain

We use a set of four physical health measures. (1) The HIV-status tested by a counsellor of the respondent coded as binary variable and available in wave six only. (2) Body Mass Index (BMI)-category of the individual coded as set of binary variables with “underweight (BMI < 18.5)”, “normal weight (18.5<=BMI<25)”, “overweight (25 < =BMI < 30)”, and “obese (BMI >=30)”, with body height and weight are measured by the interviewer in wave seven and eight. (3) Individual cognitive test score. Individuals in waves seven and eight were asked to perform cognitive tests with five tasks related to language and orientation, visual and constructional thinking, attention and working memory, executive functioning and memory. The total score of the test, ranges from 0 lowest to 30 highest. Individual cognitive skills are good predictors of mortality and show strong associations with chronic diseases [2, 4]. (4) Grip strength (in kg) averaged over the left and right hand measured in waves seven and eight. A systematic review highlights that grip strength is a valid measure of physical capability [7].

### Descriptive statistics

We focus here on the main explanatory mental health and physical variables. Table A1 in the Additional file 1 presents the descriptive statistics of all variables used in the analysis. In the 2006 MLSFH wave, 6% of respondents are tested HIV positive. Of the 2012 and 2013 MLSFH samples, 69% of individuals have normal weight, 11% are overweight and 4 % are obese. About 16% are underweight. The average cognitive test score is moderate with 20 out of 30 points. The average grip strength of both hands is 22.3 kg. Both PhQ9 and GAD7 scores are low with 2.7 and 2.4 units respectively. Individuals rate their life on average as somewhat to very satisfied.

### Statistical methods

We approach validation of the SF12 with the following five steps:

1. In line with the approach of Ware et al. [25] who developed the SF12 instruments, we use principal component analysis with the correlation matrix of the 8 sub-scale items and the standard two factor solution. The derived loadings of the first and second factor (also called component) are the weights of the 8 sub-scale items which are used to derive the respective physical and mental health dimension of the SF12 instrument. The loadings/weights are the correlation of the factor (the component) with each of the 8 sub-scale items.. We test the reliability following the standard approach in the literature by computing Cronbach’s Alpha [9, 25].
2. We apply the standard US-population weights to compute the two dimensions of the SF12 scale for the study population, leading to a Malawian-weighted and a US-weighted version of the SF12 for both health domains.
3. We use OLS regression on the 2006 and on the 2012 and 2013 survey rounds to test the construct validity for each of the four scales. We regress the four SF12 scales on the health explanatory variables and the potential confounders.
4. We use Fixed Effect regression analysis on the seventh (2012) and eighth (2013) survey round of the MLSFH to further test the construct validity to address potential unobserved time-invariant heterogeneity in mental and physical health [26].
5. We use the Malawi Incentive Programme, to identify if differences in US-weighted and Malawi-weighted SF12 mental health measures matter for estimation of policy impacts. We use SF12 outcome measures computed after the intervention had taken place in wave five (2008) of the MLSFH. First, we estimate the average effects of the cash transfer programme on mental health using both the SF12 Malawi-weighted mental health and the SF12 US-weighted mental health measure as respective outcomes. Second, we use quantile regression [8] to see if the two SF12 specifications influence the estimated impacts alongside the mental health distributions.

### Covariates

In the statistical analysis, we control for a set of mental and physical health determinants as described in Table A1. Due to missing information in the 2012 and 2013 surveys, we control in these years for a limited set of variables, which are: age, gender, ethnicity, region, educational level of the individual, his/her marital status, whether he/she lives in a house covered with a metal roof and the average number of days in a week when alcohol is consumed by the respondent.

## Results

### Weights for the SF12 - principal component analysis

Table 1 shows the correlation matrix of the eight SF12 items using Pearson correlation coefficients. We find overall high correlations between the physical and mental health items, which shows that the data is good for principal component analysis. Kaiser-Meyer-Olkin measure is 0.908 and the Bartlett test of sphericity rejects the null of no intercorrelation of variables (*p*-value: 0.000 < alpha 0.05), both indicating that the sampling is adequate to perform principal component analysis.

**Table 1:** Correlation matrix of the eight SF12 components (Pearson r)

	Physical Functioning	Role Physical	Bodily Pain	General Health	Vitality	Social Functioning	Role Emotional	Mental Health
Physical Functioning	1							
Role Physical	0.6284	1						
Bodily Pain	0.6676	0.7326	1					
General Health	0.432	0.3845	0.443	1				
Vitality	0.5914	0.6183	0.7089	0.4244	1			
Social Functioning	0.5846	0.6276	0.6913	0.3954	0.6265	1		
Role Emotional	0.3237	0.3663	0.3902	0.2519	0.3537	0.4376	1	
Mental Health	0.5121	0.5392	0.6279	0.415	0.6915	0.6814	0.5402	1

We present in Table 2 the results of the two-factor solution of the principal component analysis on the eight items of the SF12 alongside the US-population SF12 weights of the physical and mental health dimension. The first factor (component) in column one loads on all eight items of the SF12 scales and does not discriminate between the mental health items and the physical health items. The second factor in column three loads stronger on the mental health dimension and shows negative association with the items representing physical health. We report in the second and fourth column of Table 2 the weights for physical and mental health based on US-population [9]. The associations of the US-weights with the different items vary significantly compared with the association between the two factors computed using Malawian weights. Cronbach’s Alpha is 0.9 for the 8 unweighted items (sub-scales), 0.9 weighting the sub-scales with the first-factor weights (factor loadings in component 1) and 0.72 using the second factor weights (factor loadings in component 2). This indicates satisfactory reliability (> 0.7) and internal consistency of the summary scores [30].

**Table 2:** 2-factor principal component analysis for the SF12 items and SF12 US weights

SF12 components	***Component 1*** Malawi weights 1st health dimension	US Physical health weight	***Component 2*** Malawi weights 2nd health dimension	US Mental health weight
Physical Functioning	0.3578	0.42402	−0.2863	−0.22999
Role Physical	0.3717	0.35119	−0.1832	−0.12329
Body Pain	0.4003	0.31754	−0.1601	− 0.09731
General Health	0.2689	0.24954	−0.3022	−0.01571
Vitality	0.3813	0.02877	−0.0982	0.23534
Social Functioning	0.3822	−0.00753	0.0708	0.26876
Role Emotional	0.2614	−0.19206	0.8134	0.43407
Mental Health	0.3761	−0.22069	0.3022	0.48581
*Eigenvalue*	*4.7543*		*0.8293*	
*Proportion*	*0.5943*		*0.1037*	
*Observations*	*2838*		*2838*	

### Construct validity

Table 3 presents the results of the OLS-regression. HIV and subjective wellbeing both show significant associations with the first factor-weighted SF12 (model (1), Table 3). Using standardised beta-coefficients in column (2) we find that subjective wellbeing explains the main share in SF12 (0.389), four times the size of the effect of HIV. The health variables explain about 17% of the variation in SF12. The second factor weighted SF12 outcome measure is only significantly explained by subjective wellbeing in column (3). Subjective wellbeing has a negative sign. The beta-coefficients in column (4) show that HIV has a non-significant effect of almost zero (− 0.01) whereas subjective wellbeing has an effect of size − 0.168. Both variables explain only about 3% of the variation in the SF12.

**Table 3:** OLS estimation: SF12 computed on the first and second factor using the 2006 sample and the pooled 2012/13 sample

	(1)	(2)	(3)	(4)
	SF12 1st Factor	SF12 1st Factor Standardised coefficient values	SF12 2nd Factor	SF12 2nd Factor Standardised coefficient values
**Model (1) SF12 1st and 2nd Factor regressed on the Health variables using the 2006 sample**				
HIV Status	−3.583***	−0.082	−0.424	−0.010
	(1.197)		(1.217)	
Subjective Wellbeing	4.121***	0.389	−1.780***	−0.168
	(0.268)		(0.273)	
Constant	39.468***		54.007***	
	(2.176)		(2.461)	
Observations	2069	2069	2069	2069
Covariates	YES	YES	YES	YES
Region	YES	YES	YES	YES
R-squared	0.228	0.228	0.051	0.051
**Model (2) SF12 1st and 2nd Factor regressed on the Health variables using the 2012/13 sample**				
Normal Weight	0.751**	0.037	−0.497	−0.024
	(0.347)		(0.621)	
Overweight	0.557	0.019	−0.523	−0.017
	(0.484)		(0.833)	
Obese	−0.777	− 0.017	−0.314	− 0.007
	(0.714)		(1.313)	
Cognitive Test Score	0.168***	0.093	0.006	0.003
	(0.032)		(0.049)	
Average Grip Strength	0.117***	0.078	0.015	0.010
	(0.024)		(0.040)	
PhQ9 Depression Scale	−0.501***	−0.185	−0.448***	−0.162
	(0.073)		(0.108)	
GAD7 Anxiety Scale	−1.755***	−0.501	− 0.587***	− 0.163
	(0.095)		(0.141)	
Subjective Wellbeing	1.469***	0.150	− 1.727***	−0.172
	(0.140)		(0.253)	
Constant	47.889***		48.550***	
	(1.742)		(2.817)	
Observations	2091	2091	2091	2091
Covariates	YES	YES	YES	YES
Year	YES	YES	YES	YES
Region	YES	YES	YES	YES
R-squared	0.675	0.675	0.112	0.112

The outcome variable is in (1) and (2) the SF12 measure computed on the first factor loading and in (4) and (5), the SF12 measure computed on the second factor loadings for the Malawian population. In columns (2) and (4), are the standardised beta-coefficients presented. Model (1) presents findings from the analysis on the 2006 sample. Model (2) presents findings from the analysis on the 2012/13 sample. We control for year and region effects, and covariates. Robust standard errors in parentheses; *** *p* < 0.01, ** *p* < 0.05, * *p* < 0.1

In model (2), the BMI category “normal weight”, cognitive skills, grip strength, PhQ9, GAD7 and subjective wellbeing are significantly associated with SF12 weighted by the first factor in column (1). The associations with the outcome are positive for normal weight, cognitive skills, grip strength, and subjective wellbeing and negative for PhQ9 and GAD7. Mental health measures have the strongest association with SF12, with beta-coefficients of size − 0.185 (PhQ9), − 0.501 (GAD7) and 0.15 (subjective wellbeing) in column (2). The variables explain about 66% of the variation in SF12, with the majority of variance explained by mental health domain variables when estimating the model separately with mental health and physical health variables only (63.5% versus 19.6%).

Findings from the analysis using the SF12 with the second factor weights identify only significant negative associations of the three explanatory mental health variables. The explained variance is low (7.6%). When regressing the SF12 separately on physical and mental health explanatory variables, only about 0.2% of the variation in the SF12 is explained by the physical health domain variables and 7% is explained by the mental health domain variables.

Table 4 presents the findings from the OLS regression analysis using the SF12 mental and physical health dimensions computed on US-population weights. In model (1), HIV-status and subjective wellbeing are significantly associated with the SF12 outcomes in all columns with a positive sign for subjective wellbeing and a negative sign for HIV. Subjective wellbeing explains most variation in both the mental and physical SF12. The explained variation is higher for the physical health SF with 22.3% compared to 9.1% for mental health SF12.

**Table 4:** OLS estimation: SF12 computed on the US-weighting for mental and physical health using the 2006 sample and the pooled 2012/13 sample

	(1)	(2)	(3)	(4)
	SF12 PH US Covariates	SF12 PH US Standardised coefficient values	SF12 MH US Covariates	SF12 MH US Standardised coefficient values
**Model (1) SF12 PH and MH US-weighted regressed on the Health variables using the 2006 sample**				
HIV Status	−3.108***	−0.071	−2.541**	− 0.057
	(1.204)		(1.223)	
Subjective Wellbeing	4.233***	0.401	2.343***	0.219
	(0.272)		(0.263)	
Constant	39.409***		43.733***	
	(2.233)		(2.297)	
Observations	2069	2069	2069	2069
Covariates	YES	YES	YES	YES
Region	YES	YES	YES	YES
R-squared	0.223	0.223	0.091	0.091
**Model (2) SF12 PH and MH US-weighted regressed on the Health variables using the 2012/13 sample**				
Normal Weight	0.674	0.034	0.544	0.026
	(0.452)		(0.458)	
Overweight	0.112	0.004	0.884	0.029
	(0.592)		(0.645)	
Obese	−1.342	−0.029	0.170	0.004
	(0.959)		(0.988)	
Cognitive Test Score	0.118***	0.066	0.162***	0.088
	(0.038)		(0.038)	
Average Grip Strength	0.126***	0.084	0.066**	0.043
	(0.030)		(0.031)	
PhQ9 Depression Scale	−0.227***	−0.085	−0.624***	−0.225
	(0.086)		(0.084)	
GAD7 Anxiety Scale	−1.321***	−0.379	− 1.603***	− 0.445
	(0.113)		(0.110)	
Subjective Wellbeing	2.064***	0.212	0.568***	0.057
	(0.179)		(0.186)	
Constant	50.200***		45.184***	
	(2.129)		(2.155)	
Observations	2091	2091	2091	2091
Covariates	YES	YES	YES	YES
Year	YES	YES	YES	YES
Region	YES	YES	YES	YES
R-squared	0.495	0.495	0.485	0.485

The outcome variable is in (1) and (2) the SF12 US-weighted physical health measure and in (3) and (4), the SF12 US-weighted mental health measure for the Malawian population. In columns (2) and (4) are the standardised beta-coefficients presented. Model (1) presents findings from the analysis on the 2006 sample. Model (2) presents findings from the analysis on the 2012/13 sample. We control for year and region effects, and covariates. Robust standard errors in parentheses; *** *p* < 0.01, ** *p* < 0.05, * *p* < 0.1

In model (2), cognitive skills, grip strength, PhQ9, GAD7, and subjective wellbeing are significantly associated with both physical and mental health SF12. Cognitive skills, grip strength and subjective wellbeing have a positive association and PhQ9, while GAD7 has a negative association with both physical and mental SF12 measures. The GAD7 explains most of the variation with − 0.379 in physical health in column (2) and − 0.445 in mental health in column (4). The overall explained variation due to physical and mental health variables is similar for both SF12 measures: 49.5% of the physical health SF12 (column 1) and 48.5% of mental health SF12 (column 3) are explained.

Table 5 presents our findings from the Fixed Effect analysis. Columns (1) shows the results of the Malawi first factor SF12. We find that normal weight, cognitive skills and subjective wellbeing are significant and positively associated with the outcome. PhQ9 and GAD7 show a negative significant association. Mental and physical variables explain together 50% of the within individual variation, 65% of the between individual variation and 61% of the overall variation. Using separate estimation by health domain variables, 48% of the within variation, 70% of the between individual variation and 63.2% of the overall variation are explained by mental health measures. In contrast, only 4% of the within, 22.1% of the between individual and 17% of the overall variation are explained by physical health measures.

**Table 5:** Fixed Effect estimation: (1) SF12 computed on the first and second factor and (2) SF12 computed on the US-weighting for mental and physical health using 2012/13 sample

	(1)	(2)	(3)	(4)
	SF12 1st Factor	SF1s 2nd Factor	SF12 PH US	SF12 MH US
Normal Weight	1.278**	−1.003	1.748**	0.186
	(0.636)	(1.342)	(0.868)	(0.994)
Overweight	1.252	0.345	1.188	0.894
	(1.055)	(1.803)	(1.345)	(1.419)
Obese	1.849	4.104	−0.052	3.318
	(1.884)	(3.239)	(2.465)	(2.540)
Cognitive Test Score	0.212***	−0.036	0.172***	0.187***
	(0.054)	(0.084)	(0.061)	(0.068)
Average Grip Strength	0.047	0.032	0.064	0.011
	(0.045)	(0.076)	(0.052)	(0.060)
PhQ9 Depression Scale	−0.324***	−0.402***	− 0.019	−0.540***
	(0.099)	(0.154)	(0.108)	(0.121)
GAD7 Anxiety Scale	−1.662***	−0.794***	−1.238***	−1.547***
	(0.123)	(0.199)	(0.140)	(0.152)
Subjective Wellbeing	1.274***	−1.663***	1.776***	0.529**
	(0.198)	(0.348)	(0.245)	(0.270)
Constant	38.882***	−1.003	45.008***	35.619***
	(5.338)	(1.342)	(6.630)	(6.251)
Observations	2091	2091	2091	2091
Individuals	1164	1164	1164	1164
Controls	YES	YES	YES	YES
Year	YES	YES	YES	YES
Region	YES	YES	YES	YES
R-squared within	0.499	0.113	0.272	0.357
R-squared between	0.646	0.062	0.530	0.395
R-squared overall	0.605	0.068	0.462	0.378

The outcome variable is in (1) the SF12 measure computed on the first factor loading, in (2) the SF12 measure computed on the second factor loadings for the Malawian population. The outcome variable is in (3) the SF12 US-weighted physical health measure and in (4) the SF12 US-weighted mental health measure for the Malawian population. We control for year and region effects, and covariates. Robust standard errors in parentheses; *** *p* < 0.01, ** *p* < 0.05, * *p* < 0.1

Column (2) presents the results of the Malawi second factor SF12. Only the PHQ9, GAD7 and subjective wellbeing show significant and negative associations with the outcome. The overall variation explained by mental and physical health explanatory variables is 7%, within variation is 11% and between individual variation is 6%. Physical health variables alone explain only 0.04% of within individual variation, 0.02% of the between individual variation and 0% overall variation. Mental health variables explain 9.2% within individual variation, 5.8% between individual variation, and 6.7% overall variation.

Column (3) presents findings from the Fixed Effect regression with US-weighted physical health SF12. Normal weight, cognitive skills, subjective wellbeing have significant positive associations and GAD7 has a significant negative association with SF12. The health variables explain 27% of within, 53% of between individual variation and 46% in the overall variation. Compared with the physical health, the mental health measures explain more within individual physical health variation (25.7% vs. 2.9%), more between individual physical health variation (49.7 vs. 20.7%) and more overall physical health variation (42.4% vs. 15.5%).

Column (4) presents the results of the US-weighted mental health SF12. We find significant positive associations of cognitive skills and subjective wellbeing and significant negative associations of PhQ9 and GAD7 with the US-weighted mental health SF12. We find 38% of the overall variation, 36% of the within individual variation and 40% of the between individual variation explained by the health variables. Mental health variables explain more variation in the outcome than physical health variables. They explain 33.8% of the within individual (versus 2.1% for physical health variables), 53.4% of the between individual (versus 10.9% for physical health variables), and 46.6% of the overall variation (versus 7.8% for the physical health variables) in the mental health SF12.

Table A2 in the Additional file 1 presents the population norms of the SF12 measures by age-groups and gender. Mean values of the SF12 measure derived from the first factor are similar between male and females across age-groups with overlapping 95%-confidence intervals. Mean values of the instrument increase by age-groups from 49.43 in the 16–24 years age-group to 52.62 in the 55–59-years age-group. The SF12 second factor measure shows significant variation between male and females in age-groups 16–24 to 40–44 years with higher mean values for males, ranging between 51.15 (48.36) in the 40–44 age-group and 54.24 (50.39) in the 16–24 age-group for males (females). Overall, the SF12 second factor instrument decreases in age, from 51.45 in the age-group 16–24 to 46.52 in the 60+ age group.

### Application to policy evaluation

We find that different SF12 mental health measures by population weights matter for the empirical analysis. Table 6 presents in model (1) quantile and average effects of the cash transfer on mental health using the Malawi-weighted SF12 mental health measure, and in model (2) findings of the analysis using the US-weighted SF12 mental health measure. Columns (1) to (5) present the findings of the quantile treatment effect analysis for each respective quantile. Column (6) presents the average treatment effect.

**Table 6:** Quantile treatment and average effect estimation of the cash transfer on mental health using US-weights and Malawi-weights

	(1) 1st Quantile (0.1)	(2) 2nd Quantile (0.25)	(3) 3rd Quantile (0.50)	(4) 4th Quantile (0.75)	(5) 5th Quantile (0.9)	(6) Average Effect
Model (1) Estimation of quantile treatment and average effects on mental health using the Malawi-weighted SF12 mental health measure						
Treated	4.599***	1.900	0.458	0.116	0.021	1.124*
	(1.690)	(1.200)	(0.852)	(0.512)	(0.296)	(0.640)
Constant	42.259***	12.347	35.025***	43.526***	53.298***	59.915***
	(3.812)	(9.984)	(7.213)	(4.402)	(2.657)	(2.289)
Model (2) Estimation of quantile treatment and average effects on mental health using the US-weighted SF12 mental health measure						
Treated	5.305***	1.614	0.961	0.192	−0.055	1.129
	(1.696)	(1.353)	(1.163)	(0.525)	(0.082)	(0.798)
Constant	39.889***	42.957***	52.086***	56.034***	59.422***	50.014***
	(8.008)	(6.845)	(5.170)	(2.705)	(0.790)	(3.118)

The outcome variable is mental health by quantiles after the intervention for (1)–(5). The outcome variable in (6) is the change in mental health. We control for the following covariates at baseline: mental health measured by the respective SF12, membership of a local AIDS-committee, the frequency over the past months of visits to a place to see a drama, to dance, to drink beer, and to the market, self-perceived local AIDS-prevalence, probability of infant mortality, probability of a drought or equivalent food shock in the next 12 months, the number of people who have died as a result of AIDS known by the respondent, the number of funeral visits in the past month, a binary variable indicating if the individual ever smoked, one if he/she is currently smoking and one measuring the average number of days a week alcoholic drinks are consumed, a binary variable indicating if the individual lives in a house with a metal roof as a proxy for income, subjective wellbeing, a binary variable indicating the HIV-status of the individual, ethnic background (Yao, Tumbuka, Chewa or another ethnicity), educational attainment (none, primary, secondary tertiary), marital status (binary variable), the number of children living in the household, age, gender, and the number of the household members, a set of dummies for the region of origin of the respondent and a binary variable indicating if the respondent received a couple or individual cash transfer. The sample size is 790. Bootstrapped standard errors for quantiles are in parenthesis; clustered standard errors for the ITT are in parenthesis *** *p* < 0.01, ** *p* < 0.05, * *p* < 0.1. We bootstrapped the estimates on 500 repetitions

Model (1) shows significant effects of the cash transfer programme on average of size 1.1 and for the lowest mental health quantile of size 4.6, when using the Malawi-weighted SF12 mental health measure. In contrast when using the US-weighted SF12 mental health measure in model (2), we find that the cash transfer only significantly effects the lowest quantile in mental health of size 5.3 which is 15% larger than the equivalent effect in model (1). The comparison of the findings shows that the choice of SF12 measure can have significant implications for policy analysis, with significant versus non-significant average effects dependent on the specified SF12 mental health measure. We use this evidence to advocate the choice of our validated SF12 Malawian-population weighted mental health measure for future analyses. Use of US-weights can lead to different estimates of treatment effects, on average and across quantiles.

## Discussion

We computed SF12 weights for the Malawian population based on the fourth wave of the Malawian Longitudinal Study of Family and Health (MLSFH) in 2006 with a sample size of 2838 individuals. We tested and compared the content validity of our computed SF12 measures with the commonly applied US-population weighted SF12 measures using OLS and Fixed Effect regression analysis using the fourth, seventh (2012) and eighth (2013) wave of the MLSFH. We then used a Malawian cash transfer trial to test if differences between US-population weighted and Malawi population weighted SF12 measures matter for the analysis.

We find a first strong vector loading on both mental and physical health items of the SF12 scale among the Malawian population and in a second weaker vector loading on mental health variables. These findings are different to the US-population derived components which have positive loadings in physical health items and negative loadings in mental health items in the first component, and vice versa in the second component. These differences indicate that health among the Malawian population has strong interrelations between mental and physical components as opposed to the US-population. We find among all SF12 measures strongest content validity in mental health for our SF12 measure weighted by the first factor loading. None of the SF12 measures shows satisfactory properties in physical health. We find in the analysis of the cash transfer trial significant average effects of the trial on the SF12 Malawi-weighted mental health scale and no significant effects on the US-weighted mental health measure. Further the sizes of the estimated effects differ across the mental health distribution.

This is the first paper to compute SF12 health dimension weights and to test the content validity of the SF2 for a population from Sub-Saharan Africa. Another unique contribution of this paper is the use of a large representative population study with longitudinal design which permitted to analyse the temporal stability of the SF12 measures and to address unobserved heterogeneity in health, e.g. permitting to control for bias arising due past mental and physical health shocks or genetic endowment. Previous studies focused on cross-sectional design and mostly populations from western high-income countries. These studies did not find differences between SF12 measures using weights derived from western high-income samples with the common SF12 with weights computed on a US-population sample [9, 22]. A study by Patel et al. [20] tested the validity of the SF12 among a small sample of HIV positive individuals from Kenya. Like previous studies, their study used the common US-population weights in a cross-sectional design.

However, our study finds differences in the structure of the factor weights, no supporting evidence for content validity of the SF12 in physical health in general, and better construct validity for the SF12 mental health measure using weights computed on the Malawian population sample. The high percentage of explained within and between individual variation in mental health gives support for the temporal stability of the SF12 in this health dimension for the Sub-Saharan population.

A limitation of our analysis is the availability of physical health measures in the MLSFH. Whilst we use objective measures such as BMI, laboratory tested HIV-status or grip strength and cognitive test scores which are good proxy measures of physical health [2, 4, 7], future analysis should consider using alternative physical health measures such as pain scores, WHODAS, or FIM or GOS-E which can capture different physical health domains. Another limitation is that the SF12 scales are computed on the population of the 2006 sample whereas the most important validation analysis is made on an older sub-sample of the 2006 sample in 2012/2013. The benefit of this approach is the availability of clinically validated objective measures of both health dimensions in the old-age population sub-sample.

## Conclusion

Our results indicate significant differences in the construct of the SF12 measure in mental health for the population from a Low-Income Country like Malawi when using derived population weights as opposed to using US-population weights. Mental health shows more pronounced associations with the physical health dimension in the Low-Income Country. This is important for the construct validity of the SF12 health measures and for its use in impact evaluations as our health policy analysis emphasised. More research is required for the applicability of the physical health dimension of the SF12 measure in populations from Sub-Saharan Africa.

## Supplementary information

**Additional file 1: APPENDIX.** Validation of the SF12 mental and physical health measure for the population from a Low-Income Country in Sub-Saharan Africa.

## Data Availability

The datasets used for the analysis are publicly available at https://malawi.pop.upenn.edu/

## Abbreviations

BMI: Body Mass Index
GAD7: General Anxiety Disorder Assessment
MIP: Malawi Incentive Programme
MLSFH: Malawian Longitudinal Study of Aging
PhQ9: Personal Health Questionnaire 9-item version
SF12: Short Form Survey 12-item version
