# Supplementary material for: Validation of the SF12 mental and physical health measure for the population from a low-income country in sub-Saharan Africa
Source: Health Qual Life Outcomes. 2020 Mar 18;18:78. doi: 10.1186/s12955-020-01323-1 (PMC7081543; doi:10.1186/s12955-020-01323-1)
Supplement: Supplementary file 1 — Additional file 1: APPENDIX. Validation of the SF12 mental and physical health measure for the population from a Low-Income Country in Sub-Saharan Africa. [file 12955_2020_1323_MOESM1_ESM.docx]

**Appendix**: *Validation of the SF12 mental and physical health measure for the population from a low-income country in sub-Saharan Africa*

**Table A1 Descriptive statistics**

| **Variables** | **Description** | **2006 n=2,069** | **2012 n=1,034** | **2013 n=1,057** | **2012/2013 n=2,091** |
| --- | --- | --- | --- | --- | --- |
| SF12 PH US | SF12 physical health with US weights; 0 worst physical health and 100 best | 49.87 (10.129) | 50.777 (9.294) | 50.73 (9.344) | 50.75  (9.32) |
| SF12 MH US | SF12 mental health with US weights; 0 worst mental health and 100 best | 49.87 (10.30) | 50.644 (9.557) | 50.45 (9.705) | 50.546  (9.63) |
| HIV | 1 if HIV positive, 0 otherwise (VCT-counsellor tested) | 0.06 | Na. | Na. | Na. |
| Normal Weight | BMI category "normal weight" (18.5 <=BMI<25) | Na. | 0.67 | 0.7 | 0.69 |
| Overweight | BMI category "overweight" (25<=BMI<30) | Na. | 0.12 | 0.11 | 0.11 |
| Obese | BMI category "obese" (BMI>=30) | Na. | 0.04 | 0.04 | 0.04 |
| Cognitive Test Score | Cognitive skills test score, 0 worst and 30 best | Na. | 20.015 (5.224) | 20.87 (5.189) | 20.45  (5.22) |
| Average Grip Strength | Grip strength measured over left and right hand of the respondent (in kg) | Na. | 22.32 (6.30) | 22.275 (6.19) | 22.30  (6.24) |
| PhQ9 Depression Scale | Patient Health Questionnaire 9-item Depression Scale, 0 no depression and 21 highest depression | Na. | 2.89  (3.50) | 2.57  (3.437) | 2.73  (3.47) |
| GAD7 Anxiety Scale | Generalised Anxiety Disorder Assessment 7-item scale, 0 no anxiety and 21 highest anxiety score | Na. | 2.40 (2.445) | 2.412 (2.885) | 2.406  (2.675) |
| Subjective Wellbeing | 0 "Very unsatisfied" 1 "Somewhat unsatisfied" 2 "Satisfied" 3 "Somewhat satisfied" 4 "Very satisfied" | 2.98  (0.96) | 3.55 (0.999) | 3.565 (0.918) | 3.558  (0.959) |
| Female | 1 if female, 0 if male | 0.6 | 0.55 | 0.55 | 0.55 |
| Age | Age in years | 37.52 (12.79) | 58.603 (10.47) | 60.12 (10.79) | 59.37  (10.657) |
| Education | 0 "No school" 1 "Primary" 2 "Secondary" 3 "Higher" | 0.85 | 0.72 | 0.72 | 0.72 |
| Children | Number of children living in the household | 4.33  (3.433) | Na. | Na. | Na. |
| Household Size | Number of household members | 12.052 (4.093) | Na. | Na. | Na. |

Note: Descriptive statistics are on the sample of the estimated models. n indicates the number of individuals. Na.: data is not available in the survey wave. Variable means with standard errors in parenthesis.

**Descriptive Statistics continued**

| **Variables** | **Description** | **2006 n=2,069** | **2012 n=1,034** | **2013 n=1,057** | **2012/2013 n=2,091** |
| --- | --- | --- | --- | --- | --- |
| Ethnic Other | Other ethnic background | 0.14 | 0.13 | 0.13 | 0.13 |
| Ethnic Yao | Yao ethnicity | 0.24 | 0.27 | 0.27 | 0.27 |
| Ethnic Chewa | Chewa ethnicity | 0.31 | 0.27 | 0.27 | 0.27 |
| Central | Central Malawi | 0.33 | 0.3 | 0.3 | 0.3 |
| South | Southern Malawi | 0.33 | 0.35 | 0.35 | 0.35 |
| Married | 1 if married 0 otherwise | 0.92 | 0.79 | 0.78 | 0.78 |
| Metal roof | 1 if the house has a metal roof, 0 otherwise | 0.13 | 0.3 | 0.344 | 0.32 |
| Average days alcohol a week | Average number of days a week alcoholic drinks are consumed | 0.376 (0.788) | 0.384 (0.726) | 0.361 (0.676) | 0.373  (0.701) |
| Smoking | 1 if smokes, 0 otherwise | 0.16 | Na. | Na. | Na. |
| Ever Smoked | 1 if ever smoked, 0 otherwise | 0.23 | Na. | Na. | Na. |
| AIDS Committee | 1 if member of the local AIDS committee, 0 otherwise | 0.11 | Na. | Na. | Na. |
| Funeral | Number of times the individual has been to a funeral in the past month | 3.41  (2.31) | Na. | Na. | Na. |
| Visited Drama | Number of times the individual visited a drama place in the past month | 0.76  (1.82) | Na. | Na. | Na. |
| Visited Dance Place | Number of times the individual visited a dance place in the past month | 0.18  (0.86) | Na. | Na. | Na. |
| Visited Beer Place | Number of times the individual visited a beer drinking place in the past month | 0.85  (3.201) | Na. | Na. | Na. |
| Visited Market | Number of times the individual visited the market in the past month | 6.04  (6.108) | Na. | Na. | Na. |
| Number of ppl known died of AIDS | The total number of individuals known to have died of AIDS | 8.99  (8.854) | 3.879 (3.261) | 3.218 (3.195) | 3.545  (3.244) |
| AIDS Prevalence | Self-ranked AIDS-prevalence with 0 none and 10 very high prevalence | 2.934  (1.64) | Na. | Na. | Na. |
| Expected infant mortality | Perceived likelihood to die within 1 year, 0 not at all likely and 10 very likely | 2.504 (2.136) | Na. | Na. | Na. |
| Expected food shortage | Perceived likelihood of food shortage within 1 year, 0 not at all likely and 10 very likely | 4.72  (3.02) | Na. | Na. | Na. |
| Note: Descriptive statistics are on the sample of the estimated models. n indicates the number of individuals. Na.: data is not available in the survey wave. Variable means with standard errors in parenthesis | | | | | |

**Table A2 Population norms SF 12 measures by gender and age-groups**

|  |  |  | **Malawi SF12 1^st^ Factor** | | |  | **Malawi SF12 2^nd^ Factor** | | |
| --- | --- | --- | --- | --- | --- | --- | --- | --- | --- |
| **Age group (years)** | **Gender** | **n** | **Mean** | **SD** | **[95% CI]** |  | **Mean** | **SD** | **[95% CI]** |
| 16-24 | Male | 147 | 48.85 | 8.06 | [47.54-50.16] |  | 54.24 | 6.13 | [53.24-55.24] |
|  | Female | 384 | 49.66 | 9.80 | [48.67-50.64] |  | 50.39 | 9.38 | [49.45-51.33] |
|  | All | 531 | 49.43 | 9.35 | [48.64-50.23] |  | 51.45 | 8.77 | [50.71-52.2] |
| 25-29 | Male | 145 | 49.35 | 8.59 | [47.94-50.76] |  | 52.84 | 7.07 | [51.68-54] |
|  | Female | 269 | 49.18 | 10.68 | [47.9-50.46] |  | 50.17 | 9.35 | [49.05-51.29] |
|  | All | 414 | 49.24 | 9.99 | [48.27-50.2] |  | 51.10 | 8.71 | [50.26-51.95] |
| 30-34 | Male | 150 | 49.37 | 7.59 | [48.15-50.6] |  | 52.27 | 7.57 | [51.04-53.49] |
|  | Female | 243 | 49.38 | 10.97 | [47.99-50.77] |  | 49.42 | 10.82 | [48.05-50.79] |
|  | All | 393 | 49.38 | 9.81 | [48.4-50.35] |  | 50.51 | 9.79 | [49.54-51.48] |
| 35-39 | Male | 176 | 49.41 | 8.29 | [48.18-50.64] |  | 52.46 | 7.58 | [51.33-53.58] |
|  | Female | 193 | 50.08 | 9.79 | [48.69-51.47] |  | 49.24 | 10.59 | [47.73-50.74] |
|  | All | 369 | 49.76 | 9.10 | [48.83-50.69] |  | 50.77 | 9.41 | [49.81-51.73] |
| 40-44 | Male | 107 | 48.69 | 10.54 | [46.67-50.71] |  | 51.15 | 9.70 | [49.29-53.01] |
|  | Female | 158 | 51.11 | 10.64 | [49.44-52.78] |  | 48.36 | 11.11 | [46.61-50.11] |
|  | All | 265 | 50.13 | 10.64 | [48.85-51.42] |  | 49.49 | 10.63 | [48.2-50.77] |
| 45-49 | Male | 125 | 49.84 | 9.03 | [48.25-51.44] |  | 51.37 | 8.46 | [49.88-52.87] |
|  | Female | 130 | 49.85 | 11.88 | [47.79-51.91] |  | 48.78 | 9.63 | [47.11-50.45] |
|  | All | 255 | 49.85 | 10.56 | [48.54-51.15] |  | 50.05 | 9.15 | [48.92-51.18] |
| 50-54 | Male | 106 | 49.70 | 11.27 | [47.53-51.87] |  | 50.74 | 8.43 | [49.12-52.37] |
|  | Female | 87 | 52.18 | 10.39 | [49.97-54.4] |  | 44.73 | 14.07 | [41.73-47.72] |
|  | All | 193 | 50.82 | 10.92 | [49.27-52.37] |  | 48.03 | 11.68 | [46.37-49.69] |
| 55-59 | Male | 76 | 53.12 | 7.54 | [51.4-54.84] |  | 50.45 | 9.18 | [48.35-52.54] |
|  | Female | 44 | 51.76 | 11.09 | [48.39-55.14] |  | 43.12 | 13.94 | [38.88-47.36] |
|  | All | 120 | 52.62 | 8.98 | [51-54.25] |  | 47.76 | 11.66 | [45.65-49.87] |
| 60+ | Male | 109 | 51.88 | 11.29 | [49.74-54.02] |  | 46.32 | 12.99 | [43.85-48.78] |
|  | Female | 66 | 50.81 | 9.10 | [48.57-53.04] |  | 46.86 | 11.88 | [43.94-49.78] |
|  | All | 175 | 51.48 | 10.50 | [49.91-53.04] |  | 46.52 | 12.55 | [44.65-48.4] |
| Total | Male | 1,193 | 49.87 | 9.15 | [49.35-50.39] |  | 51.61 | 8.62 | [51.12-52.09] |
|  | Female | 1,643 | 50.08 | 10.54 | [49.57-50.59] |  | 48.95 | 10.67 | [48.43-49.46] |
|  | All | 2,836 | 49.99 | 9.98 | [49.63-50.36] |  | 50.06 | 9.94 | [49.7-50.43] |
| Note: SF12 Mental Health and Physical Health Malawian weighted instruments population norms by age-group and gender with means, standard deviations (SD) and 95%-Confidence Intervals in brackets. | | | | | | | | | |
